# Tympanoplasty: Surgical results and a comparison of the factors that may interfere in their success

**DOI:** 10.1016/S1808-8694(15)30067-7

**Published:** 2015-10-19

**Authors:** Ilana Fukuchi, Dafne Patrícia Cerchiari, Eduardo Garcia, Carlos Eduardo Borges Rezende, Priscila Bogar Rapoport

**Affiliations:** a MD, Resident in Otorhinolaryngology - Medical School of the ABC.; b MD, Resident in Otorhinolaryngology - Medical School of the ABC.; c 5th year medical student - Medical School of the ABC.; d MD, Otorhinolaryngologist, Assistant Professor - Department of Otorhinolaryngology - Medical School of the ABC.; e PhD in Otorhinolaryngology - USP, Full Professor of Otorhinolaryngology - Medical School of the ABC.

**Keywords:** chronic otitis media, tympanoplasty

## Abstract

Chronic otitis media has a high prevalence on the population and their treatment continuous to be a challenge for the otorhinolaryngologists. **Aim:** To demonstrate the factors that could interfere in the tympanoplasty success and the surgical results during 2002. **Study Design:** Clinical prospective. **Material and Method:** were included 37 patients with chronic otitis media non cholesteatoma (COMNC) undergo to tympanoplasty (in lay or underlay, with homologous graft). All the patients were submitted to a survey pre and postoperative include clinical, physical examinations, flexible nasal endoscope and audiometry. **Results:** The age, the dimension and localization of the tympanic membrane perforation; the condition of middle ear mucosa; number of otorrhea/year; smoking; parents history of otorrhea and hearing loss; personals history of otological surgery; monthly family income; the graft, technique and access used were not significantly to repair tympanic membrane perforations. The closure rate was 65% and the gain in air-bone gap was 100%. **Conclusion:** The timpanoplasty must be considerate in the treatment of the COMNC.

## INTRODUCTION

Otitis media (OM) is a worldly prevalent disease. Despite all scientific progress (antibiotics, technology and knowledge), OM remains as an important public health problem, since otalgia, discomfort, hearing loss, otorrhea, psychological trauma and complications cause great personal and family suffering^1^.

OM may be defined as a focal or generalized inflammatory process with or without infection, in the auditory apparatus. According to Bluestone and Kenna (1988), otitis media may be divided in acute or chronic, suppurative otitis media and non-suppurative otitis media, further broken down into serous and secretory^2^, ^3^.

Chronic Otitis Media is clinically characterized as an inflammatory condition associated to broad and persistent tympanic membrane perforations and otorrhea. Histologically it may be defined as an inflammatory process of the middle ear associated to irreversible tissue alterations. It may be further broken down into non-cholesteatomatous chronic otitis media (NCCOM) and cholesteatomatous chronic otitis media (CCOM). The difference between these two groups is the presence or absence of a cholesteatoma^1^.

The major symptom the NCCOM patients present is intermittent otorrhea, usually associated to upper airway infections or a past history of extrinsic contamination (swimming in pools or ocean), painless, without giving off foul smell, together with hearing loss. By doing otoscopy we usually find a perforation in the pars tensa of the tympanic membrane of varied size and shape, the middle ear mucosa has an almost normal appearance, except for some degrees of hyperemia^1^.

The hearing loss associated to this pathology is conductive and may vary considerably. Some co-factors that influence the severity of such hearing loss are the size and position of the tympanic perforation, the degree of membrane and ossicles fixation, ossicle erosions, ossicular chain disruption and the degree of repercussion in the inner ear^1^.

NCCOM treatment and control are imperative because of the auditory sequels and morbidity. It involves three equally important and complementary stages: preoperative clinical control, surgical treatment and post-operative follow up^1^, ^4^.

Pre-operative clinical treatment is based on the removal of all epithelial residues as well as all secretions from the middle and outer ear, use topical drops with acidifying agents and antibiotics; ear protection; control allergic rhinitis and control the factors that may prevent the proper functioning of the Eustachian tube^1^.

Surgery is based on tympanoplasty with middle ear and ossicular chain exploration, and tympanic membrane reconstruction. It aims at reestablishing sound protection obtaining a cavity filled with air and restoring the mechanisms that transmit sound, improving hearing and stopping otohrea^4^.

Post-operative follow up has to be thorough, with ear protection and prohibition of physical activities and Valsalva maneuvers; maintain good nasal breathing, oral antibiotics and ear drops, besides return for clinical control. Audiogram should be performed after the third month of post-op.

The goal of the present study is to show the surgical and audiometric results of patients with non-cholesteatomatous otitis media who underwent tympanoplasty surgery during the year of 2004 and analyze the factors that may have influenced surgery success.

## MATERIALS AND METHODS

We included 37 patients with NCCOM seen at the Otorhinolaryngology ward of the Medical School of the ABC Medical School, who underwent tympanoplasties from March 2004 to December of the same year.

All the patients who presented signs and symptoms suggesting NCCOM were submitted to an assessment protocol, supervised by the same examiner (C.E.B.R.), based on a guided history taking, specific physical exam (otoscopy and rhinoscopy), audiogram, nasofibroscopy and video documentation through video-otoscopy to better visualize the tympanic membrane perforation.

During history taking, the patients were questioned about disease onset, period of time spent without otorrhea, number of otologic infections per year and if they had undergone previous otologic surgeries. Besides, the patients were questioned about family income, whether or not there was family history of hearing loss and otorrhea; and vices, such as smoking. Nasal complaints were also relevant.

Otoscopic assessment considered perforation size (percentage of area perforated in the tympanic membrane), location according to quadrant (antero-inferior, antero-superior, postero-inferior and postero-superior), tympanosclerosis, possibility of visualizing all the perforation borders and presence or absence of inflammatory mucosa in the middle ear.

All patients underwent flexible nasofibroscopy (model Pentax 3,7mm) in order to check nasal conditions, looking for pathologies that could interfere in the proper functioning of the Eustachian tube.

The audiometric exam comprised vocal and tonal audiogram in the following frequencies: 250, 500, 1000, 2000, 3000, 4000, 6000 and 8000Hz in all the patients who presented satisfactory clinical control (no otorrhea for at least four months), and they were the candidates for the surgical procedure.

All procedures were carried out under general anesthesia, using a microscope with a lens of 250mm, by the second year residents of the Mário Covas State Hospital - Santo André-SP, supervised by the same instructor (C.E.B.R.).

The in-lay technique was used for perforations smaller than 40% of total membrane area and the underlay approach for larger perforations; the perforation was approached in a transmeatal via when the perforation borders were visible and the external acoustic meatus favored its visualization, and the perforation was approached via retroauricular incision when one of the borders was not seen or the external acoustic meatus was too curved and thus preventing the visualization of the whole perforation; as to the type of graft, we used either the temporal muscle fascia or tragus cartilage with its pericondrium, the latter is the best option for the in-lay technique.

The transmeatal technique followed the following surgical steps: (1) infiltrating the posterior wall of the external acoustic meatus with xylocaine and adrenalin 1: 40.000 solution; (2) scraping of the perforation borders and removal of tympanosclerosis plates; (3) longitudinal incision in the meatus at 6 and 12 o’clock positions; (4) joining the vertical incisions through a transversal incision at 5 to 7 cm away from the tympanic membrane; (5) detachment and pushover of the tympanic-meatal flap; (6) filling the tympanic cavity with Gelfoam® up to the perforation height; (7) graft placement underneath the tympanic remains, adjusting its borders to the inside of the perforation; (8) filling the external acoustic meatus with Gelfoam® and gauze soaked in antibiotic ointment; (9) external dressing.

For the retroauricular via, our steps are as follows: (1) infiltration with xylocaine + adrenalin 1: 40.000 in the retroauricular region; (2) skin and subcutaneous incision in the retroauricular groove, detachment of the periosteum flap all the way to external acoustic meatus; (3) incision in the external acoustic meatus skin in the osteocartilaginous junction; (4) we repeat the steps from 2 to 8 in the transmeatal via; (9) suture by planes; (10) external dressing.

The in-lay technique was the following: (1) scraping the borders of the tympanic perforation through the canal; (2) graft preparation with tragus cartilage and pericondrium forming a sort of a reel; (3) graft placement as we place a ventilation tube.

Hospital stay is of one day, using IV analgesic and antibiotics, keeping oral medication for one week after hospital discharge.

Post-operative follow up happened with patient returns after 3, 7, 15, 30, 60, 90 and 120 days. The first audiogram was ordered 90 days after surgery and questioned as to functional improvement.

If the perforation recurred, the patient was offered the same procedure and follow up aforementioned.

Statistical evaluation was carried out through chi-squared, t test, Mann-Whitney and Wilcoxon tests for a pre and postoperative comparison of the parameters studied.

## RESULTS

Of the total 37 patients with NCCOM who underwent tympanoplasty, 57% were women and 43% men. Average age was of 25 ± 10 years (13 to 56 years).

As to the perforation side, 65% (24 patients) had it on the right side and 35% (13 patients) on the left side. The perforations were more often located on the antero-inferior quadrant (75.7%), followed by postero-inferior (59.5%), antero-inferior (13.5%) and postero-inferior (2.7%). The perforation size was in average 40.9%.

Most patients presented more than 5 ear infections per year (54%) and no infection was mentioned by 27% of the patients. Only 10 (27%) patients had tympanosclerosis and most of them (89.2%) had non-hypoplasic mucosa in the tympanic cavity.

As to previous history, seven patients (18.9%) reported family history of hearing loss and 14 patients (37.8%) reported episodes of family otorrhea. Five patients (13.5%) had already been operated at least once because of the same problem.

In 30 patients (81%) was possible to visualize all the borders of the perforation, and this was also the number of patients who underwent transmeatal tympanoplasty. Temporal muscle fascia was used as graft in 27 patients (73%), and in 10 patients (27%) we used cartilage from the tragus.

Table 1 depicts both the technique and the type of graft used for each patient who obtained tympanic membrane perforation closure.

**Table 1:** Relationship between the technique and the type of graft used in those patients which had total repair of their tympanic membrane.

Via	Transmeatal		Retroauricular
Technique/ Graft type	in-lay	underlay	underlay
Fascia	0	9	4
Cartilagem	6	0	0
Total	6	9	4

Eight patients (216%) had postoperative infection.

Surgery success, considering tympanic membrane perforation closure happened in 19 patients (51.4%). Considering the re-operations carried out after the beginning of this protocol, this number went up in 24 patients (65%). In all the patients that had perforation repair there was auditory gain, proven by the audiogram and the average gain was of 18.7 dBHL. Of the 19 patients who had successful closure of their tympanic membrane perforation in the first surgery, 14 patients (74%) had their postoperative hearing gap merged.

Most of our patients did not have alterations under nasofibroscopy (70.3% - 26 patients); of the 11 patients who had nasal complaints, five (13.5%) underwent septoplasty and bilateral partial inferior turbinectomy before the ear surgery; one patient (2.7%) suffered adenoidectomy and the other five (13.5%) patients improved with the use of nasal topic steroids and systemic antihistamine.

The different factors studied that could impact surgery success are depicted on Table 2. The technique, the access and the type of graft used in the surgery were not statistically significant for the perforation closure. The perforation site in the different quadrants was also not statistically significantly.

**Table 2:** Average of the factors that could influence or not the tympanic membrane perforation repair.

	Age	T wt/otor	T ds	Inf/year	% perf	Income/m	GAP pre	GAP pos
PR	26,39	42,06	12,33	4,17	37,22	734,72	26,11	23,89
NPR	25,11	9,90	11,57	5,26	43,16	573,68	21,05	2,37
p statistic	0,165	0,001*	0,631	0,703	0,539	0,388	0,800	0,002*

PR: perforation repair/ NPR: no perforation repair/ T wt/otor: Time without otorrhea in months/ T ds: Disease time in years/ Inf/year: infections per year % perf: percentage of perforation/ Income/m: monthly income in reals/ GAP pre: difference between bone conduction and air conduction in tonal audiometry in the preoperative / GAP pos: difference between bone conduction and air conduction in tonal audiometry in the postoperative

* Values statistically significant by the T test.

Being a smoker or not, having a family history of otorrhea and hearing loss, past surgery, presence of neotympanic membrane and non-hyperplasic tympanic cavity mucosa were not statistically relevant in this group studied.

As to perforation size, most of the patients (30 patients - 81%) had size reduction, 5 patients had size increase (13.5%) and 2 patients (5.4%) kept the same perforation they had in the preoperative.

## DISCUSSION

The procedures that aim at repairing the tympanic membrane have always been considered as the easiest ones in otology. Success rates reported in the literature have always been optimistic. In order to matching such expectations, the authors of the present study aimed at providing a realistic view of tympanoplasties carried out in modern times^8^.

The major goals of tympanoplasty are to reduce the number of infections and improve hearing. Success rates vary in the literature, from 75 to 98%. In our study we found a success rate of 65%, a below the average value considering the repair of tympanic membrane perforations^5^.

Bhat e Ranit (2000) reported that the factors that may influence surgery success rates are: age, perforation location and size, Eustachian tube conditions, status of the middle ear mucosa, type of graft used and surgeon experience^5^.

Age is not a factor that alters tympanoplasties success rates. The most important step for indicating surgery in the elderly population is a preoperative care, assessing nutritional status, cardiovascular condition, metabolic status and mental condition^6^, and also a more rigorous anesthetic evaluation. As far as children are concerned, the restrictions are the same as those for adults, with special attention given to the children psychological profile, which should follow rigorously the advices of rest and ear protection, parents’ consent, enough age for proper mastoid bone, Eustachian tube and immune system development^7^. The population of the present study varied in age from 13 to 56 years; therefore in this sample we had no representatives from the geriatric and pediatric populations, thus not being possible to correlate age as a significant success factor for surgery.

Anterior perforations represent a worse surgical access in order to reach the anterior border, and they are also less vascularized, because of that they are considered an important success factor for surgery. Many authors believe that the perforation location plays a more important role in surgery success than perforation size^5^, ^8^.

The functional role of the Eustachian tube is still not well defined^9^.

The middle ear mucosal status suggests disease activity. Thus, if there is mucosal hyperplasia, this may mean poor aeration of the middle ear, suggesting disease activity. Therefore, studies show that with a minimum interval of three months without otorrhea, the surgery success rate increases to more than 30% when compared to the cases that underwent surgery in an infected site^8^.

In our study, parameters such as perforation size and location, Eustachian tube status, middle ear mucosa status, type of graft used and others hereby mentioned, such as disease development, number of infections per year, percentage of membrane involved in the perforation, or monthly income did not prove to be statistically important for obtaining surgical success.

The main grafts used are the tragus cartilage pericondrium, vein walls, dura mater, fascia lata, free skin graft and temporal muscle fascia. The temporal muscle fascia is preferred for being of easier access, possibility of using the same incision (in some cases) and for bearing low metabolic index^5^. Currently, the perforations that involve up to 40% of the tympanic membrane and bear low level hearing loss, tragus cartilage has shown great effectiveness.

The type of graft used, as well as the surgical technique used, had no statistical significance for the surgical success in the patients studied. The low success rate of 51.4% (19 patients) found in our study in the first surgery may be linked to graft atrophy, since 18 patients (48.6%) did not have their perforations closed, 8 patients (44.4%) presented signs of infection in the postoperative. Of these 18, only 8 patients were reoperated until the present date, and of these, 5 (63%) obtained success. Of the three who were not successful, two patients reported upper airway infections in the first week of postoperative and one patient exercised on the first week of post op.

Tympanoplasties aim at repairing tympanic membrane perforations and obtain audiometric gain. In this study we observed a hearing gap reduction in most of the sample patients (34 patients - 92%). Only 3 patients (8%) had a hearing gap enlargement after the first surgery, they were reoperated and had a gap reduction. Thus, considering hearing improvement, we may suggest that all patients benefited from surgery.

We believe that this hearing improvement is due to the fact that there was a reduction in perforation size in most of the patients studied.

## CONCLUSION

The factors considered by many authors as relevant for the tympanic membrane repair were not statistically significant in our study.

The audiometric gain was found in most of the patients after the first surgery and in 100% of the patients after reoperations.

As to tympanic membrane perforation repair, the low success rate found in this study (65%) may be attributed to the surgeon since all surgeries were by performed by second year residents.

## References

[1] Costa SS, Sousa LCA, Campos CAH, Costa HOO (2002). Tratado de Otorrinolaringologia.

[2] Costa SS (1991). Dissertação (Mestrado). Faculdade de Medicina de Ribeirão Preto.

[3] Bluestone CD, Kenna MA (1988). Workshop on chronic suppurative otitis media. Ann Otol Rhinol Laryngol.

[4] Cruz OLM, Costa SS, Kluwe LH, Smith MM, Cruz OLM, Costa SS (2000). Otologia Clínica e Cirúrgica.

[5] Bhat NA, Ranit De (2000). Retrospective Analysis of Surgical Outcome, Symptom Changes, and Hearing Improvement Following Myringoplasty. J Otol.

[6] Emmett JR (1996). Age as a factor in the success of tympanoplasty: A comparison of outcomes in the young and old. Am J Otol.

[7] Berger G, Berger S (2002). Paediatric revision myringoplasty: outcomes and prospects. J Laryngol Otol.

[8] Gersdorff M, Garin P, Decat M, Juantegui M (1995). Myringoplasty: long-term results in adults and children. Am J Otol.

[9] Gimenez F, Marco-Algarra J, Carbonell R, Morant A, Cano S. (1993). Prognostic factors in tympanoplasty: a statistical evaluation. Rev Laryngol.

